# Psychological Capital in Food Safety Social Co-governance

**DOI:** 10.3389/fpsyg.2019.01387

**Published:** 2019-07-01

**Authors:** Xiujuan Chen, Linhai Wu

**Affiliations:** Institute for Food Safety Risk Management, School of Business, Jiangnan University, Wuxi, China

**Keywords:** psychological capital, food safety, social co-governance, industry, government

## Abstract

Social co-governance of food safety is an advocacy model to deal with current global food safety risks. Food safety co-governance involves the collaboration and partnership of government, industry, and society. The success of this collaboration is dependent on the construct of positive psychological capital. This paper discusses the concept of psychological capital and its four elements of self-efficacy, optimism, hope, and resiliency in line with co-governance in food safety. Accordingly, the paper proposes that great success in food safety co-governance would be realized if the government, industry, and society nurture positive psychological capital. Psychological capital can help corporates to instill organizational commitment on employees, thus promote food safety. Furthermore, positive psychological capital can help the government to appeal to the emotions of food companies and social actors to ensure self-efficacy toward food safety. The government can inspire hope by setting food safety goals and plans to achieve them, and a reward program will motivate food companies and promote self-efficacy in co-governance efforts. The government can also reinforce the efforts of companies in leveraging the elements of PsyCap in food safety, since the production of high-quality food is also dependent on the behaviors and attitudes of the workforce. Furthermore, the government can utilize social persuasion to improve the engagement of social actors in food safety regulations.

## Introduction

Food safety is a challenge that governments across the globe are grappling with, particularly in developing countries. According to Wu et al. (2018), establishing a better and sustainable food economy requires the integration of diverse efforts and resources from various stakeholders. The ideal of social co-governance involves core elements of legal protection, public participation, social cooperation, government regulation, and corporate self-governance (Wu et al., 2018). This paper further discusses the concept of psychological capital (PsyCap) and its four elements of self-efficacy, optimism, hope, and resiliency in line with co-governance in food safety. Accordingly, the paper proposes that great success in food safety co-governance would be realized if the government, industry, and society nurture positive psychological capital.

Social co-governance is a concept developed with the purpose of enhancing collaboration and partnership between the government, industry, and the citizens. The main agenda behind co-governance is to reduce costs while improving the efficacy of food safety regulations. Involving all the actors in co-governance helps to enhance the practicality of decisions made and to reduce the burden on the players (Wu et al., 2018). Nevertheless, co-governance alone is not enough without leveraging the role of the PsyCap in the decision making processes. While the government plays a regulatory role on the actors in the food safety industry, these regulations may become futile if the four elements of psychological capital are ignored. Furthermore, since food industry players may be incentivized by the government to maintain food safety, these private-sector organizations may not achieve maximum potential if they have not instilled the construct of psychological capital in their workforce. Likewise, the public may not appreciate the efforts of the government and the industry and thus may not exude commitment, fulfillment, or satisfaction in the efforts toward food safety.

PsyCap forms the basis for thriving and wellbeing as it ignites positive emotions and a sense of appreciation. The realization of these positive emotions depends on the four pillars of PsyCap that are hope, optimism, self-efficacy, and resilience. Hope involves a positive motivation from a goal-directed drive combined with planning to meet the goals. Hope inspires one to set goals and strive to achieve them (Steeneveld, 2015). Optimism involves a great personal ability and sense of confidence when dealing with problems. Optimism instills great faith in one’s abilities to improve situations. The concept of self-efficacy refers to one’s belief in their skills to accomplish set targets effectively. Parker (1998) suggests that self-efficient employees exude confidence to perform wide-ranging roles in an organization. Lastly, resilience encompasses the ability to “bounce back and beyond” in the face of adversities (Masten, 2001). Resilience enables one to cope and thrive in difficult situations. Professor Luthans (2002) sums up the aims of positive psychology as shifting emphasis from negative to positive perspectives about people, shifting from weaknesses to strengths, focusing more on resilience than vulnerability, and directing efforts and resources to enhancement and development of wellness, prosperity, and the good life rather than remediating on pathology. According to Luthans et al. (2006), PsyCap is playing a very critical role in shaping positive organizational behavior (POB).

## Role of Psychological Capital in Government Governance

Food safety is a public problem that involves multi-stakeholder input from the government, industry, and the public. The government formulates and implements regulations on food safety. While there may be a sound regulatory framework, the public may be reluctant to follow the rules where there is no motivation to do so. When leading in the midst of a crisis, it is essential for the government to leverage PsyCap to appeal to the public emotionally. For instance, during World War II, Winston Churchill used emotional appeal to engage the citizenry and to instill the belief in them that their efforts and resources were not hopeless (Milosevic et al., 2017). World War II is one of the most fatal crises in history as it resulted in more than 60 million casualties (Kershaw, 2007). Throughout this period, Churchill utilized positive psychological capital in fueling the acquisition of resources and steering activities to navigate the crisis. According to Milosevic et al. (2017), Churchill instilled hope in the people by formulating clear goals and confidence by developing a belief for the realization of the goals, regardless of the many obstacles that lay before him. In the Battle of Britain, which was the first critical event for Britain in the World War II, Churchill leveraged psychological capital to mobilize resources and to emotionally appeal to the people for purposes of replenishing depleted armies.

In the second major event of World War II, that is, the Attack on Pearl Harbor, the United States joined the war. At this point, Church focused on leveraging the values platform required to develop and nurture positive relationships with the Allies who had different political ideologies with Britain. Churchill utilized all the four elements of PsyCap to achieve the desired positive relationships. Finally, in the Battle of Stalingrad, the positive emotions and vigor illustrated by the people and the armies propelled Europe to optimize on the significant losses of German in the East and emerge victoriously. Churchill knew that to defeat Germany, Britain could not succeed in working in isolation. There was a need to developed ties across the “whole world” to fight “evil-doers.” Churchill managed to leverage the PsyCap to imbue his followers into a belief that regaining independence from Germany was the only way of defeating German armies (Milosevic et al., 2017). He instilled the spirit of hope, optimism, self-efficacy, and resilience in the Britons and people across the country, thus leading to the acquisition of more resources and human capital needed for the war.

When the government formulates rules on food safety, it is not enough to impose them on the people. Instead, it is essential for the leadership to establish whether the people are willing to follow the rules. Accordingly, the government must determine whether the food industry and social actors are eager to support the regulatory framework on food safety. According to Baker et al. (2015), the tendency to follow the leader is a form of deference whereby the followers choose to take part in leadership processes actively. In this regard, leadership can be successful where the leader does not understand the followers and their behaviors (Uhl-Bien et al., 2014). Churchill leveraged the PsyCap to imbue his followers into action and lead the whole continent out of war regardless of the scarce resources available to his disposal (Milosevic et al., 2017).

In practice, the government primarily focuses on enhancing regulatory standards on food safety with prominence on severe punishment to non-compliant actors. Due to public pressure from a series of food safety incidents, many developed countries have sought to strengthen their food safety risk governance through strict legislation and direct intervention involving severe punishments. Nevertheless, problems still arise from the absolute reliance on the administrative agencies to implement food safety standards. Regardless of the available regulatory framework, food companies remain compliant to the regulations basing on a cost-benefit analysis. Furthermore, food companies decide on which rules to follow depending on whether they can navigate around external incentives and internal resources – human capital. For most companies, producing high-quality food is not merely to evade punishment but to maintain a good reputation in the society and thus reap great benefits. Social actors, on the other hand, are deemed as a powerful tool for supplementing the governance by the government and self-governance by the corporates. Social actors include citizens and various social organizations such as self-help groups, foundations, advocacy groups, social service agents, educational institutions, health-care organizations, clubs, and associations. Since social actors are on the receiving end of food safety, they stand out as the best regulators. However, without information on food safety, the public may be limited to effectively participate in food safety co-governance (Wu et al., 2018). Thus, the regulatory capability of consumers can become more effective if food safety systems operate in a transparent and traceable manner.

According to Luthans et al. (2013), positive psychological capital can extend beyond organizational contexts into other spheres of life. Following a decade of research and theory building, PsyCap currently applies across the world towards decisive leadership (Youssef and Luthans, 2012). In this regard, leveraging positive psychological capital in food safety co-governance will guarantee greater success for all the actors. The government can harness the PsyCap in food safety co-governance by using the Winston Churchill approach to appeal to the emotions of the food companies and social actors. The government can inspire hope by setting food safety goals and plans to achieve them. Rather than only focusing severe punishment to corporate actors for non-compliance with the regulations, the government can set up measures for rewarding food companies which accomplish the set targets. A reward program will motivate food companies and promote self-efficacy in co-governance efforts.

Furthermore, the goals will be more realistic to the industry actors if they are well-aligned to their corporate objectives for profit-making. Besides, food companies will be more willing to follow regulations which are clearly communicated to them and do not significantly jeopardize organizational goals. Since the production of high-quality food is also dependent on the behaviors and attitudes of the workforce, the government can also reinforce the efforts of companies in leveraging the elements of PsyCap in food safety.

## Role of Psychological Capital in Corporate Co-Governance

According to Milosevic et al. (2017), leaders in an organization must learn to deal with a crisis in today’s world. Osborn et al. (2002) describe a crisis as a “dramatic departure from prior practice and sudden threats to high priority goals with little or no response.” Accordingly, positive psychological capital can be useful for organizational leaders in navigating a crisis (Avey et al., 2011). Furthermore, research indicates that PsyCap can allow organizations to navigate challenging circumstances due to the motivation to achieve success (Luthans and Youssef, 2007). PsyCap drives leaders to focus on motivating effort and perseverance by positively appraising conditions, thus enabling organizing to navigate a crisis and develop strong determination towards the future.

Psychologists relate the four components of PsyCap to higher job commitment, performance, and satisfaction particularly in the service industry (Luthans and Youssef, 2007; Abbas et al., 2014). According to Avey et al. (2011), higher PsyCap results in organizational citizenship behaviors, higher job commitment and satisfaction, lower intentions to quit, and lower absenteeism. Furthermore, there is an essential relationship between the levels of PsyCap and desirable employee attitudes, behaviors, and performance (Abbas et al., 2014). Leaders can utilize the concept of PsyCap to explore the untapped territories of human strengths, excellence, and wellbeing (Luthans and Youssef, 2007).

Making PsyCap as a foundation in the food industry can tremendously impact on the efforts of co-governance toward food safety. The human capital in the food industry plays a vital role in food safety. McMahon (2007) notes that most food processing companies envision organizational commitment from their employees. Accordingly, any given organizational context can leverage organizational commitment, job satisfaction, and work engagement. Employee commitment enables an organization to thrive and succeed in the wide-ranging and dynamic business environment.

Furthermore, companies across sectors are increasingly focusing on developing their human capital as a way of achieving competitive advantage (Adler and Kwon, 2002). According to Luthans and Youssef (2007), psychological capital is the most tangible and ultimate resource in the changing business environments. Psychological capital tends to be more important than human capital and social capital in the food industry.

Whereas social capital involves who one knows and human capital involves what one knows, psychological capital centers on who one is and who one is developing into, thus forming the basis of the current self and possible self (Gota, 2017). Some scholars argue that organizations which focus on attracting, engaging, developing, and retaining the brightest talent have the most robust human capital (O’Leary et al., 2002). Other scholars suggest that connections, networks, and social relations are the most critical capital for the organization as they help to leverage more information and lead to greater influence and control power (Adler and Kwon, 2002). Nevertheless, social and human capital is not enough to reap optimal performance for the organization. An organization needs to tap into psychological capital to maintain a competitive advantage. This argument derives from the fact that PsyCap pushes one to ask the question “who am I?” (Luthans and Youssef, 2007), which improves one’s self-awareness as a fundamental basis to leadership development and influence. Thus, PsyCap makes a food company first explore its position in the market and to further develop strategies in line with its status as perceived by employees, consumers, and the government. Furthermore, the importance of psychological capital in food safety is because developing the psychological capital of employees has the potential of improving their performance (Luthans et al., 2006).

Furthermore, an organization that promotes collective PsyCap can tremendously increase team performance (Toor and Ofori, 2009). Since employees with high psychological capital are more productive in an organization, food industry leaders can capitalize on this to promote food safety. According to Luthans et al. (2005), positive PsyCap management has the abilities to leverage the strengths, talents, and potentials of employees to realize a long-term competitive advantage. Organizations can achieve this through periodical evaluation of employees for psychological capital and providing them with strength-based feedback for purposes of building positive capabilities.

In regard to work engagement, food processing companies can work on providing an enabling environment to employees as well as a supportive culture to promote high performance. Promotion of personal fulfillment alongside organizational success requires organizations to align the individual goals of their employees with corporate goals (Luthans et al., 2013). Workforce efforts are likely to complement smart goals of the organization. The food industry organizations can successfully meet their part in the co-governance in food safety by assisting employees to develop smart goals with the aim of directing the behavior of employees and establishing deliberate work behaviors such as organizational commitment and work engagement (Davids, 2011, unpublished; Levene, 2015). Furthermore, it is essential for organizations to leverage positive psychological capital to attract, develop, and retain employees with an avid commitment to ensuring food safety. Food industry companies can formulate and implement human resource development practices with the aim of up-skilling their workforce to promote organizational commitment to food safety (Gota, 2017). Furthermore, food processing companies can enhance organizational commitment to food safety by leveraging organizational goals with the specific needs and expectations of employees.

A significant character of PsyCap is that that it is dynamic (Luthans et al., 2013). More specifically, the elements of PsyCap are “state-like” in the sense that they are malleable and more open to change and development than the “trait-like” attributes of the Big Five personality constructs. Accordingly, psychological capital is a uniquely versatile and dynamic resource, which enables leaders to leverage their PsyCap to enhance the PsyCap of the collective. Thus, transformational leaders have the abilities to use PsyCap in fueling powerful visions and influencing followers to establish and implement positive goals, pursue positive future expectations, instill strong beliefs of self-efficacy in the followers, and enable followers to endure through a crisis. Furthermore, an ethical leader is capable of channeling high levels of PsyCap towards beneficial outcomes.

## Role of Psychological Capital in Social Actors Co-Governance

It may tend to appear a “double failure” of government public right and market private right in the process of food safety risk management. Thus, social actors are needed to participate in the food safety social co-governance. Social actors are important players in a country’s food safety governance. In the task of creating a pattern of food safety co-governance, the active and effective participation of social actors is very critical. Whether social actors can play their unique advantages in the food safety social co-governance and make up for the double failure of food safety risks, depends to a considerable extent on the external policy environment in which social actors are located (Li et al., 2016).

With regard to the social actors, the government can promote co-governance by creating an enabling environment for them to engage in food safety regulation actively. Engaging the public in food safety requires the government to establish common ground in line with the four elements of PsyCap. According to Ohlin (2017), it is possible to attain efficacy through a mastery of experiences, social modeling, social persuasion, and psychological responses. The government can work towards increasing awareness of the strengths, traits, and factors that resulted in the success. These efforts should be combined by the government and the industry to share relevant information with social actors, which can form the basis for the society to appraise best performing food companies. In this line, the government will be reinforcing self-efficacy for the food companies while also appealing to the positive emotions of the society. Social modeling can also be used to achieve self-efficacy by the government developing models for food safety from companies that have overcome obstacles. Social modeling can encourage food companies to benchmark with the best ranking companies with the aim of promoting their reputation (Ohlin, 2017). Furthermore, the government can utilize social persuasion to improve the engagement of social actors in food safety regulations.

Therefore, it is necessary to fully recognize the role of PsyCap in stimulating the vitality of social actors, and make full use of PsyCap to promote social actors to participate in food safety social co-governance. From the perspective of PsyCap, social actors, as beneficiaries of food safety, have a strong motivation to improve food safety and ensure their own health. The government can stimulate the hope of social actors by setting food safety goals and plans, and create positive food safety governance environment to ignite the positive emotions and feelings of appreciation of social participants, so as to ensure that social actors can improve their self-efficacy in participating in food safety co-governance.

China is the largest developing country in the world. Generally speaking, the role of social actors in China’s food safety social co-governance is still limited at this stage. For example, our previous study found that the public’s willingness to participate in food traceability information query is seriously insufficient (Chen et al., 2018). If the Chinese government wants to effectively implement the food traceability system in the whole society, which is a public system of food safety, on the one hand, it needs the cooperation of enterprises; on the other hand, it cannot do without the participation of social actors. Considering the role of PsyCap, we suggest that the government disseminate the food safety traceability concept, cultivate the traceability culture, and form a social atmosphere that is familiar with traceability, supports traceability, and actively participates in traceability, to enhance the social actors’ self-efficacy, optimism, and positive emotions. This is an example of the use of PsyCap to improve the participation of social actors in food safety social co-governance.

## Conclusion

Food safety has been the center of social discussion in all cultures across the world. To address the many challenges associated with ensuring food safety, one discipline, such as food science, managerial science, or economics is not sufficient, and it is difficult to effectively promote food safety relying on just one actor’s effort, such as government, corporate, or social actor. A cross-disciplinary and pluralistic actors approach should be adopted, which points to the need of societal co-governance. Social co-governance of food safety has become an advocacy model to deal with the current global food safety risks and challenges. Food safety social co-governance involves the collaboration and partnership of government, industry, and society. The success of this collaboration is dependent on the construct of positive psychological capital, which comprises hope, self-efficacy, resilience, and optimism. These four elements form the basis for effective co-governance. In practice, the government primarily focuses on enhancing regulatory standards on food safety with prominence on severe punishment to non-compliant actors. Food processing companies, on the other hand, focus on making profits, while social actors keep looking for loopholes and pushing for food safety. Positive psychological capital can help the government to appeal to the emotions of food companies and social actors to ensure self-efficacy toward food safety. Furthermore, PsyCap can help corporates to instill organizational commitment on employees, thus promote food safety.

The concept of PsyCap and its four components listed are new in the literature on food safety social co-governance. This paper may be the first to introduce PsyCap into food safety co-governance, providing a useful and timely discussion on means to facilitate such co-governance. It proposes a fresh perspective that great success in food safety co-governance would be realized if the government, industry, and society nurture positive psychological capital. This current work comes from the angle of psychological research thus limiting itself to a more qualitative analysis but the discussions offered good inspiration and guidance for theoretical thinking and practical imperatives.

As this study is an enlightening conceptual study, no empirical data are provided. In the future studies, we will conduct empirical survey, for example, on the social co-governance of abuse of food additives in processed food in China, by interviewing government regulators, food enterprises, social organizations and the public to collect data. Through the empirical analysis, we aim to explore main factors of PsyCap that influence different players involved in food safety social co-governance, to provide useful suggestions for the government to improve the social environment. We also hope there will be more empirical research samples from different countries and regions on the application of PsyCap in the field of food safety social co-governance, so as to carry out comparative studies and provide more research data for this topic.

## Data Availability

All datasets analyzed for this study are cited in the manuscript and the supplementary files.

## Author Contributions

XC and LW both contributed to the development of the theme and writing the manuscript.

### Conflict of Interest Statement

The authors declare that the research was conducted in the absence of any commercial or financial relationships that could be construed as a potential conflict of interest.
